# Particle therapy in Europe

**DOI:** 10.1002/1878-0261.12677

**Published:** 2020-04-22

**Authors:** Cai Grau, Marco Durante, Dietmar Georg, Johannes A. Langendijk, Damien C. Weber

**Affiliations:** ^1^ Department of Oncology and Danish Center for Particle Therapy Aarhus University Hospital Aarhus Denmark; ^2^ Biophysics Department GSI Helmholtzzentrum für Schwerionenforschung Darmstadt Germany; ^3^ Institut für Festkörperphysik Technische Universität Darmstadt Germany; ^4^ Department of Radiation Oncology Medical University of Vienna/AKH Wien Vienna Austria; ^5^ Department of Radiation Oncology University Medical Centrum Groningen Groningen The Netherlands; ^6^ Paul Scherrer Institute Villigen Switzerland

**Keywords:** cancer, ion beam therapy, particle therapy, proton therapy, radiation, radiotherapy

## Abstract

Particle therapy using protons or heavier ions is currently the most advanced form of radiotherapy and offers new opportunities for improving cancer care and research. Ions deposit the dose with a sharp maximum – the Bragg peak – and normal tissue receives a much lower dose than what is delivered by X‐ray therapy. Particle therapy has also biological advantages due to the high linear energy transfer of the charged particles around the Bragg peak. The introduction of particle therapy has been slow in Europe, but within the last decade, more than 20 clinical facilities have opened and facilitated access to this frontline therapy. In this review article, the basic concepts of particle therapy are reviewed along with a presentation of the current clinical indications, the European clinical research, and the established networks.

## Graphical abstract

Particle therapy using protons, or heavier ions, is the most advanced form of radiotherapy today and offers new opportunities for improving cancer care and research. Within the last decade, more than 20 new clinical facilities have opened in Europe, facilitating access to this frontline therapy. This review presents the physics, biology, and clinical aspects of particle therapy.

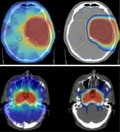

**Abbreviations**
CT: computer tomography
EORTC: European Organization for Research and Treatment of Cancer
EPTN: European Particle Therapy Network
ESTRO: European Society for Radiotherapy and Oncology
LET: linear energy transfer
MeV·u^−1^: energy range per nucleon
MR: magnetic resonance imaging
NTCP: normal tissue complication probability
PET: positron emission tomography
PTCOG: Particle Therapy Co‐Operative Group
RBE: relative biological effectiveness

## 1 Introduction

Particle therapy denotes the clinical use of ion beams (protons or heavier ions) to treat patients with malignant or nonmalignant tumors. Particle therapy is currently the most advanced form of radiotherapy. Despite a somewhat higher cost compared to megavoltage X‐rays, ion beam therapy holds great potential for significant advances in cancer care and research. Within the last decade, more than 20 clinical facilities have opened in European countries, which has enabled easier access to this frontline therapy also for European citizens.

**Fig. 1:**
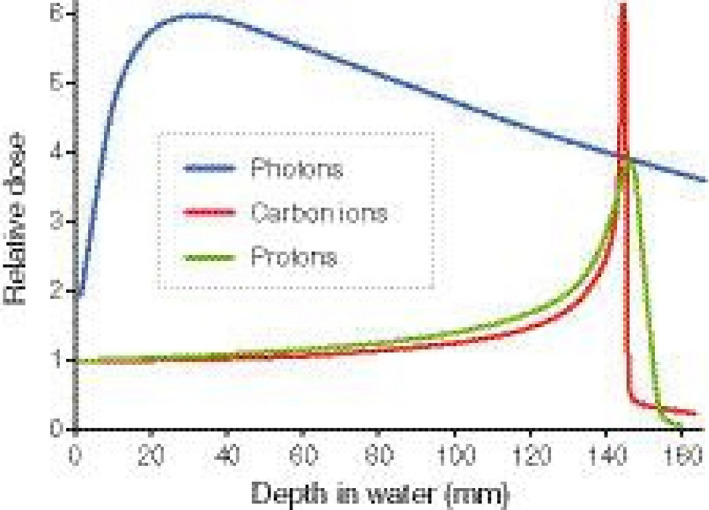
Physical and biological advantages of particle therapy (protons and carbon ions) as compared to megavoltage X‐rays (photons). The depth–dose curves of charged particles are defined by a plateau phase and the Bragg peak, situated in a specific depth depending on the energy of the beam.

The application of accelerated charged particles in radiotherapy was originally proposed in 1946 by Robert Rathnub Wilson, a student of Ernest Orlando Lawrence at the University of California in Berkeley (CA, USA) (Wilson, 1946). The physical advantages of particle therapy are due to the favorable depth–dose distribution compared to conventional X‐rays (Durante and Paganetti, 2016). While photon dose decreases exponentially as a function of the depth, swift ions deposit initially little energy (plateau), but their energy loss per unit track increases with depth, reaching a sharp maximum, called the Bragg peak, when they are close to their range in tissue (Fig. 1). Therefore, the dose to the organs at risk can be reduced with ion beam without compromising the dose to the tumor. In photon radiotherapy, it is necessary to combine many different beam angles, whereas only a few beams are necessary in ion beam therapy (Durante and Paganetti, 2016).

In addition, particle therapy has several biological advantages compared to conventional photon radiotherapy (Durante and Loeffler, 2010). The advantages are due to the high linear energy transfer (LET) of the charged particles around the Bragg peak: The dense ionization patterns induce clustered DNA lesions that are difficult to repair and trigger different signaling pathways. The effectiveness of high‐LET radiation in tumor control was the main rationale for using fast neutrons in radiotherapy (Specht *et al.*, 2015), but in neutron therapy, the LET is high in both normal and tumor tissues, and the depth–dose distribution is similar to photons. On the other hand, in particle therapy the LET can be low in the entrance (plateau) and high in the Bragg peak, and the high‐LET radiobiological properties are therefore confined to the tumor region (Fig. 1). Because LET ∝*z* ^2^, where *z* is the atomic number of the ion, heavy ions have higher LET than light ions, but for *z* ≥ 10, the LET is too high already in the normal tissue, thus leading to unacceptable toxicities such as those observed in neutron therapy. For these very reasons, particle therapy is limited to light ions, and currently, only protons and ^12^C‐ions are used in the clinics.

Protons (*z* = 1) have always low LET, and therefore, their advantage is limited to the sparing of the normal tissue due to the Bragg peak. However, it has been recently observed that the increased LET of slow protons at the end of their range can lead to unexpected toxicities, such as brain necrosis (Haas‐Kogan *et al.*, 2018). Carbon ions (*z* = 6) are a good compromise because they have low LET in the entrance channel and high LET in the tumor region (Durante and Debus, 2018). For this reason, carbon ion therapy is currently ongoing in nine centers in Asia and four in Europe (Weber *et al.*, 2017). However, carbon ion therapy is more expensive than proton therapy, because large synchrotrons are needed to accelerate heavy ions compared to compact cyclotrons used in proton therapy (Durante and Flanz, 2019).

The aim of the current paper is to introduce the basic concepts of particle therapy, including the biological properties and the technical and physical aspects of various delivery techniques, along with a review of the contemporary clinical use of protons in Europe. Finally, the European networks for experimental and clinical research will be described.

## 2 Ion beam delivery techniques for clinical use

The energies required in proton beam therapy range from about 60 to 250 MeV to cover penetration depth from a few centimeters (e.g., for ocular tumors) to more than 25 cm (e.g., for pelvic tumors). For heavier particles such as carbon ions, the respective energy range per nucleon (MeV·u^−1^) is between about around 120 and 430 MeV·u^−1^ (Stock *et al.*, 2018). Most European particle therapy facilities that focus on proton therapy use cyclotrons for beam production, while the four European particle therapy facilities that offer both protons and carbon ions are synchrotron‐based. A cyclotron is operated at a static magnetic field; hence, the radius of the beam cycling in the magnetic field increases with increasing particle beam energy. The beam is extracted from the cyclotron when it reaches the maximum energy and is reduced passively by inserting range shifters in the beam line to reduce penetration depth. In a synchrotron, the particle beam is cycling through a ring with a fixed radius at variable magnetic field strengths to compensate for the particle energy. The beam is incrementally accelerated and can be extracted from the ring at any energy. This allows to actively select beam energies instead of degrading a high energy by inserting passive elements.

Irrespective of accelerator type, after beam extraction the narrow particle beam (‘pencil beam’) is transported through vacuum pipes into the treatment room. In order to shape the narrow beam to an irregular tumor, two main delivery techniques are in clinical use, that is, the passive scattering and pencil beam scanning technique. In the traditionally used passive scattering technique, the pencil beam is widened by mechanical elements that scatter the beam; field shaping according to the 2D projection of the tumor is achieved by individual collimators. The depth–dose modulation of the Bragg peak to cover the tumor extent in longitudinal direction is also realized via passive elements. All newly planned and recently established particle therapy facilities apply or aim for pencil beam scanning, where the narrow PB is deflected by magnetic fields in vertical and horizontal directions. The position in depth of the Bragg peak is adjusted via active energy variations in case of synchrotrons, or by reducing the maximum particle energy with range shifter in case of cyclotrons. As the scanning technique does not need mechanical elements for beam broadening or beam shaping, which cause unwanted secondary particles (mostly neutrons), it results in a lower integral dose to healthy tissue (Schneider *et al.*, 2016). Two European particle therapy facilities, the Paul Scherrer Institute in Villigen, Switzerland, for proton therapy (spot scanning) and the Helmholtzzentrum für Schwerionenforschung in Darmstadt, Germany, for carbon ion therapy (raster scanning), pioneered scanned beam delivery and thus paved the way for its global clinical utilization.

In order to detect, assess, delineate, and track the tumor volume with the highest possible precision, imaging plays a vital role in particle therapy. Irrespective of beam delivery technique, treatment planning is commonly based on multimodality imaging (CT, MR, PET). The pencil beam scanning technique is linked with computerized treatment plan optimization, where the particle fluence pattern to be delivered is calculated from desired treatment aims, that is, the intended target dose and the acceptable organ at risk exposure (Oelfke and Bortfeld, 2003). Treatment planning for the traditional passive scattering technique is performed in a manual trial and error optimization process.

Influenced and motivated by the standards of image‐guided photon beam therapy, the integration of advanced X‐ray imaging (e.g., cone‐beam computed tomography) in the treatment room has just started in particle therapy (Bolsi *et al.*, 2018). In‐room imaging enables to account for time variable anatomic variations during the course of therapy. Moreover, frequent and repetitive imaging is the prerequisite for adaptive radiotherapy, where patient‐specific variations that were detected via imaging methods trigger treatment modifications (van de Water *et al.*, 2018). Treating moving targets with particle therapy is challenging and certainly needs an image‐guided approach (Knopf *et al.*, 2010). In the context of motion mitigation techniques like gating or tumor tracking, the time structure of the beam produced by either a synchrotron or a cyclotron is important as well.

## 3 Clinical experience and current indications

**Fig. 2:**
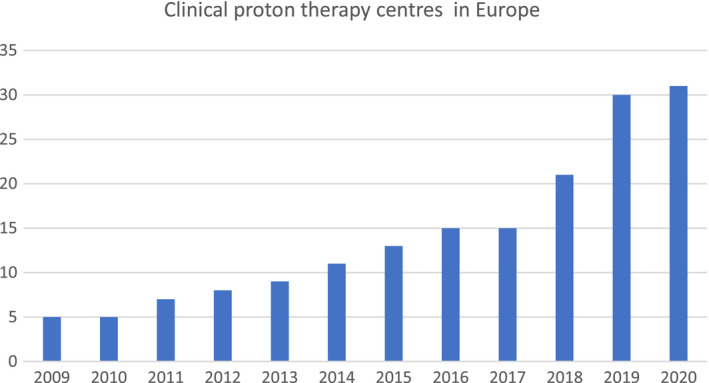
Graph showing the number of clinical proton facilities in Europe 2009–2020. Source: www.ptcog.ch.

It took only 6 years after the publication by Robert R. Wilson in 1946 before John Lawrence and Cornelius A. Tobias at Berkeley Radiation Laboratory applied protons clinically for the first time when a 340‐MeV proton beam was used to irradiate the pituitary gland in a patient with acromegaly. Since then, more than 190 000 patients have been treated with protons and 28 000 patients have received carbon ions (www.ptcog.ch). Eighty proton therapy centers are currently active worldwide; about 30% of those centers are in Europe. The number of European proton therapy clinics is rapidly increasing: While in 2017 there were 15 operational facilities, by the end of 2020 there will be 31 proton therapy facilities in clinical operation (Fig. 2). Carbon ion therapy is currently ongoing in nine centers in Asia and four in Europe.

**Fig. 3:**
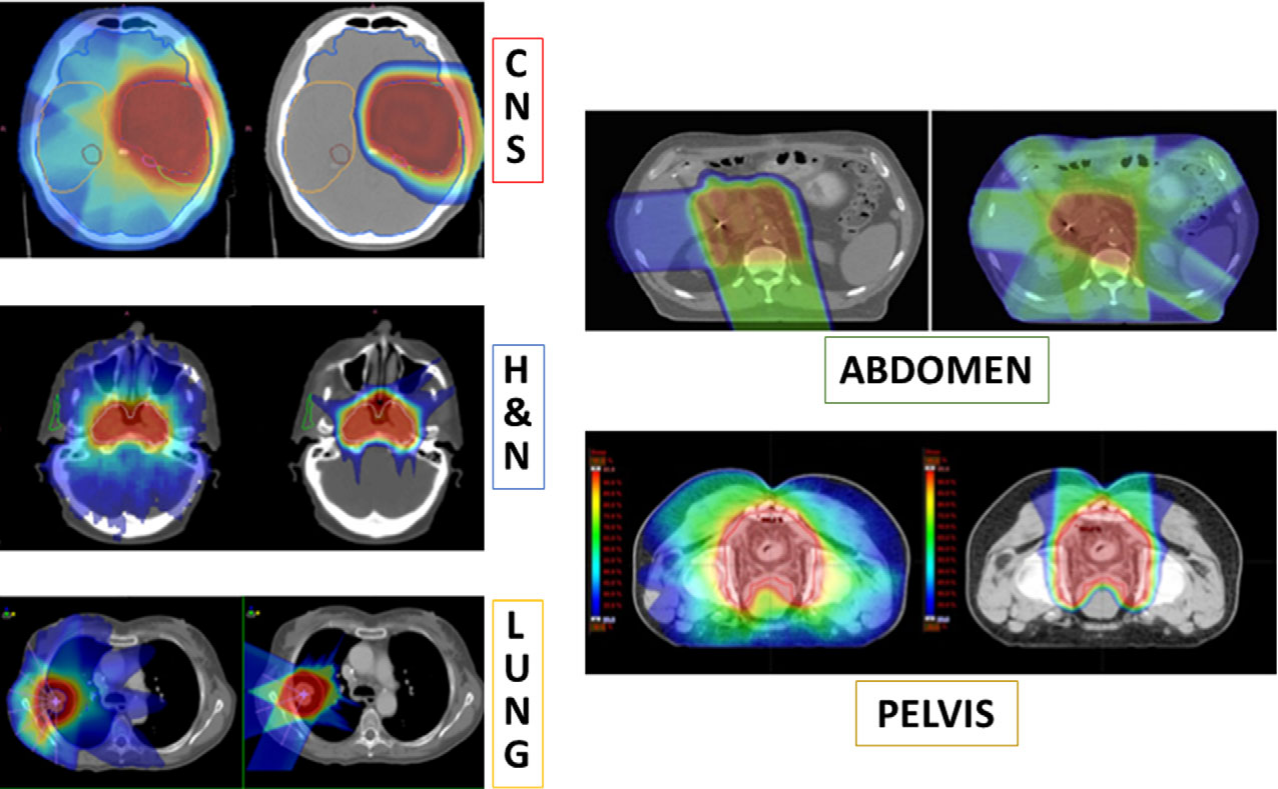
Comparison of treatment plans with X‐rays (left) and protons (right) for different tumor sites. Figure reproduced from Durante *et al.* (2019), reproduced with permission of Elsevier.

The clinical rationale for using proton therapy is primarily based on the advantageous physical dose distribution. Protons can be used to increase the target dose, as to optimize local tumor control probability, and/or to decrease the likelihood of radiation‐induced toxicity by delivering less dose to organs at risk in the direct vicinity of the tumor volume (Fig. 3). Skull base tumors (i.e., chordoma or chondrosarcoma), which are radioresistant tumors, are good examples where tumor control is increased by a factor of approximately three to four when using protons as compared to historical photon series (Romero *et al.*, 1993; Weber *et al.*, 2016). Reduction of radiation‐induced toxicity is seen, for example, in medulloblastoma in children, wherein the radiation dose delivered outside the target volume in the patient's body is reduced by 4.5–6.0 times with proton therapy as compared to photon therapy, resulting in a substantial risk of radiation‐induced secondary malignancies (Sakthivel *et al.*, 2019).

Proton therapy is currently considered standard therapy only in a limited number of cancer types. A current list of core indications is detailed in Table 1. The list may vary from country to country. In Europe, such indications are generally negotiated on a national level. Most European countries accept proton therapy as a standard for pediatric indications. For all other indications, some countries reimburse proton therapy according to a binding list (e.g., France, Italy, Poland, Switzerland). Other countries (e.g., Sweden, Denmark, the Czech Republic) do not have a fixed list of approved indications; here, the administration of proton therapy is based on decisions from multidisciplinary tumor boards. One country (the Netherlands) applies the model‐based approach (Widder *et al.*, 2016) for selected novel indications (see the next section). In many countries, including Germany and the United States, cancers are treated with proton therapy providing that the patient's healthcare package reimburses this treatment. In the United States, a substantial number of patients however have been refused proton therapy (Odei *et al.*, 2017; Shen *et al.*, 2017).

**Table 1:** Core indications for proton therapy.

Ocular tumors
Uveal, iris, and conjunctival melanoma; hemangioma
Skull base tumors
Primary skull base tumors
Secondary infiltration from intracranial tumors
Head and neck tumors
Nasopharyngeal carcinoma, paranasal sinus carcinoma, adenoid cystic carcinoma, parotid carcinoma, soft tissue sarcomas
Nonmoving extracranial (paraspinal, retroperitoneal, sacral) tumors
Chondrosarcoma, chordoma, osteosarcoma, soft tissue sarcoma
Intracranial and spinal tumors
Low‐grade glioma, ependymoma, medulloblastoma, meningioma, chordoma, chondrosarcoma, neuroblastoma, low‐grade glioma NOS
Tumors of pediatric patients
The indications listed above and other pediatric tumors, for example, rhabdomyosarcoma, primitive neuroectodermal tumors, atypical teratoid/rhabdoid tumors, and Ewing sarcoma

The clinical rationale for using carbon ion therapy is related to the depth–dose characteristics, a sharper penumbra, and the biological effectiveness, which is greater than that of protons. Carbon ions are used only in clinical protocols, and for certain types of, for example, radioresistant cancer.

## 4 Randomized clinical trials and new evidence‐based methodologies

Although particle therapy has been clinically introduced already for decades for several indications, limited data exist on treatment outcome based on systemically collected prospective studies, such as randomized controlled trials or observational cohort studies. Since particle therapy is more expensive than conventional photon radiotherapy, it is of utmost importance that robust data are generated to justify its use (Grau *et al.*, 2018); evidence from well‐designed prospective trials is needed to demonstrate that protons delivered to selected cancer patients have a meaningful clinical benefit and should be routinely administered to a broader range of patients (Langendijk *et al.*, 2018a; Weber *et al.*, 2019, 2020).

There are currently a number of prospective phase III randomized trials ongoing or in the late planning phase in Europe. Most advanced are the plans for comparing protons with photons (IMRT) for head and neck cancers. In the UK, a randomized trial (TORPEdO) in oropharyngeal cancer patients has been launched (Price *et al.*, 2020). In Denmark, the Danish Head and Neck Cancer Group (DAHANCA) is preparing a trial in oropharyngeal and laryngeal cancers, where patients who have a dosimetric benefit of protons will be randomized to either modality. EORTC is considering a trial, which in addition to the randomized arms allows patients to be enrolled using a model‐based approach without randomization. Other European randomized trials are under way in breast cancer, esophageal cancer, and prostate and lung cancers. These trials will form a much‐needed balance to a large series of US trials already recruiting.

Next to the randomized controlled trial, there is a need for alternative evidence‐based methodologies. In the Netherlands, the so‐called model‐based approach has been introduced not only to select which patients likely benefit most from proton therapy (model‐based selection), but also to define optimization criteria for radiotherapy treatment planning based on dose parameters included in normal tissue complication probability (NTCP) models (model‐based optimization) and model‐based clinical validation (Christianen *et al.*, 2016; Langendijk *et al.*, 2018a, 2013, 2018a, 2013; Rwigema *et al.*, 2019; Widder *et al.*, 2016). Prospective data registries are the backbone of the model‐based approach and are essential to continuously develop, externally validate, and update NTCP models for photons and protons. However, the model‐based approach is only applicable when protons are used to decrease the dose to critical anatomical structures aiming at reduction of radiation‐induced side effects, while the target dose remains biologically equivalent as compared to photons (Langendijk *et al.*, 2013). Consequently, the model‐based approach cannot be used to validate the added value of carbon ions, assuming a relative biological effectiveness (RBE) that is different from photons, and/or if particles are used for target dose escalation. Moreover, the model‐based approach assumes that high‐quality NTCP models are available, which is not always the case for all indications. Therefore, in these circumstances, randomized controlled trials remain the gold standard.

Other novel trial methods are currently being suggested, many of which utilize information from rigorously collected prospective clinical data. One of these, the so‐called cohort multiple randomized controlled trial, will be described in the next section.

## 5 Prospective clinical databases

Prospective real‐life data registries may provide important information on the outcome of patients treated with particle therapy, both in terms of efficacy (e.g., local control and survival) and in terms of side effects and patient‐rated outcome measures. As such, prospective registrations offer a unique opportunity to find out which factors in the care process lead to the best results for patients. Identifying variation between centers and sharing positive results and care processes of best practices may give major incentives to improve the quality of all particle therapy centers or to follow implementation of guidelines (Beck *et al.*, 2019; Gietelink *et al.*, 2016).

The European Society for Radiotherapy and Oncology (ESTRO) is setting up a prospective data registration program for patients treated with particle therapy in the ParticleCare project (Weber *et al.*, 2019). ParticleCare is part of E^2^‐RADIatE (www.estro.org/Science/E2RADIATE), which stands for the EORTC‐ESTRO RADiotherapy InfrAstrucTure for Europe, a collaboration between the European Organization for Research and Treatment of Cancer (EORTC) and the European Society for Radiotherapy. The overarching aim of ParticleCare is to establish a uniform prospective data registry on a European level for the most common tumor types treated with particle therapy, such as for cancers of the central nervous system, head and neck, breast, lung, esophagus, and prostate (Langendijk *et al.*, 2018b).

Prospective data registries are an essential component of the so‐called cohort multiple randomized controlled trials (Lambin *et al.*, 2015; Langendijk *et al.*, 2018a). The principle is that a large cohort of patients is monitored prospectively for various parameters (Relton *et al.*, 2010). Compared to a classical randomized trial, the main difference is that the study population can be randomly assigned to one of several new interventions (e.g., protons instead of photons) when they meet specific conditions. In this way, several novel approaches can be tested in the same trial. The trial design is particularly appropriate when testing expensive interventions like protons, and in situations where patients' preference to accept a new intervention is high (Lambin *et al.*, 2015).

## 6 Collaborative networks

Collaboration between European particle therapy centers for generation of scientific and clinical evidence is of critical importance (Weber *et al.*, 2019).

European particle therapy centers are active members of the global Particle Therapy Co‐Operative Group (PTCOG; www.ptcog.ch). In addition, there are currently three active European networks working in complementary fields of particle therapy: the European Network for Light Ion Hadron Therapy (ENLIGHT; https://enlight.web.cern.ch), the Infrastructure in Proton International Research (INSPIRE; www.protonsinspire.eu), and the European Particle Therapy Network (EPTN; www.estro.org/Science/EPTN). In addition, research collaboration is facilitated through the International Biophysics Collaboration (www.gsi.de/bio‐coll) and an EU collaboration exists to support particle therapy in southeastern Europe (www.seeiist.eu). The EU also sponsored a previous collaboration, the Union of Light Ion Centres in Europe (ULICE; www.cordis.europa.eu/project/rcn/92176/reporting/en). The ULICE project ended in 2014, leaving a substantial contribution of reports and white papers in the public domain (Potter *et al.*, 2018).

The ENLIGHT network has coordinated European efforts in ion beam research since 2002. The network has its main focus on science dissemination within basic and translational research issues (Dosanjh *et al.*, 2018a).

INSPIRE was funded by EU FP7 for the period of 2018–2021. A broad consortium led by the University of Manchester works together to develop a new infrastructure, bringing research activities in clinical proton therapy centers, associated academic establishments, and industry across Europe together. The aim is to enable researchers from across Europe, in both the public and private sectors, to access this infrastructure and conduct research. Training will be provided for the next generation of researchers in this field where there is an internationally recognized skill shortage. INSPIRE will also facilitate knowledge exchange and allow best research practice to be shared across and between centers throughout Europe and develop an innovation pipeline allowing research to be translated into clinical practice and industrial products (www.inspireprotons.eu).

The EPTN was established in 2015 in response to the increase in the number of clinical particle therapy centers in Europe (Grau *et al.*, 2018; Weber *et al.*, 2019). The primary aim of EPTN is to enable cooperation among particle therapy centers and to integrate particle therapy in the radiation oncology community (Grau *et al.*, 2018). Therefore, EPTN is an official task force of the European Society for Radiotherapy and Oncology (ESTRO). The seven working groups have already produced a series of recommendations and whitepapers, and more activities are expected in the coming years (Bolsi *et al.*, 2018; Dosanjh *et al.*, 2018b; Eekers *et al.*, 2018; Grau *et al.*, 2018; Lambrecht *et al.*, 2018; Langendijk *et al.*, 2018b; Weber *et al.*, 2019, 2018, 2019, 2018).

## 7 Conclusions and future directions

Particle therapy holds great promise to improve the therapeutic outcome of cancer patients treated with this modality. There is an urgency to produce high‐quality clinical evidence. With the opening of a substantial number of particle centers in Europe, there is now a great opportunity to collaborate across institutions and countries to secure evidence‐based implementation of particle therapy. In Europe, collaboration is catalyzed by several networks and organizations, including INSPIRE, EPTN, ESTRO, and EORTC. There is now a platform that enables European particle centers to collaborate in prospective data collection as well as prospective clinical trials. With the combined efforts of all these dedicated working groups and institutions, there is no doubt that European particle therapy is ready to meet the challenge and generate the much‐needed clinical evidence for particle therapy.

## Conflict of interest

The authors declare no conflict of interest.

## Author contributions

All authors contributed to the manuscript and approved the final version.
